# Roles of TRPV_1 _and neuropeptidergic receptors in dorsal root reflex-mediated neurogenic inflammation induced by intradermal injection of capsaicin

**DOI:** 10.1186/1744-8069-3-30

**Published:** 2007-10-25

**Authors:** Qing Lin, Dingge Li, Xijin Xu, Xiaoju Zou, Li Fang

**Affiliations:** 1 Department of Neuroscience and Cell Biology, The University of Texas Medical Branch, Galveston, Texas 77555-1069, USA; 2 Division of Neurosurgery, Department of Surgery, University of Texas Medical Branch, Galveston, Texas 77555-1043, USA; 3 Division of Neurotoxicology, National Center for Toxicological Research, Food and Drug Administration, 3900 NCTR Road, Jefferson, Arkansas 72079-9502, USA

## Abstract

**Background:**

Acute cutaneous neurogenic inflammation initiated by activation of transient receptor potential vanilloid-1 (TRPV_1_) receptors following intradermal injection of capsaicin is mediated mainly by dorsal root reflexes (DRRs). Inflammatory neuropeptides are suggested to be released from primary afferent nociceptors participating in inflammation. However, no direct evidence demonstrates that the release of inflammatory substances is due to the triggering of DRRs and how activation of TRPV_1 _receptors initiates neurogenic inflammation via triggering DRRs.

**Results:**

Here we used pharmacological manipulations to analyze the roles of TRPV_1 _and neuropeptidergic receptors in the DRR-mediated neurogenic inflammation induced by intradermal injection of capsaicin. The degree of cutaneous inflammation in the hindpaw that followed capsaicin injection was assessed by measurements of local blood flow (vasodilation) and paw-thickness (edema) of the foot skin in anesthetized rats. Local injection of capsaicin, calcitonin gene-related peptide (CGRP) or substance P (SP) resulted in cutaneous vasodilation and edema. Removal of DRRs by either spinal dorsal rhizotomy or intrathecal administration of the GABA_A _receptor antagonist, bicuculline, reduced dramatically the capsaicin-induced vasodilation and edema. In contrast, CGRP- or SP-induced inflammation was not significantly affected after DRR removal. Dose-response analysis of the antagonistic effect of the TRPV_1 _receptor antagonist, capsazepine administered peripherally, shows that the capsaicin-evoked inflammation was inhibited in a dose-dependent manner, and nearly completely abolished by capsazepine at doses between 30–150 μg. In contrast, pretreatment of the periphery with different doses of CGRP_8–37 _(a CGRP receptor antagonist) or spantide I (a neurokinin 1 receptor antagonist) only reduced the inflammation. If both CGRP and NK_1 _receptors were blocked by co-administration of CGRP_8–37 _and spantide I, a stronger reduction in the capsaicin-initiated inflammation was produced.

**Conclusion:**

Our data suggest that 1) the generation of DRRs is critical for driving the release of neuropeptides antidromically from primary afferent nociceptors; 2) activation of TRPV_1 _receptors in primary afferent nociceptors following intradermal capsaicin injection initiates this process; 3) the released CGRP and SP participate in neurogenic inflammation.

## Background

The inflammation initiated by release of inflammatory mediators from primary afferent nerve terminals (mainly nociceptors) is referred to as neurogenic inflammation [1,2]. A wide range of inflammatory diseases like allergic arthritis, asthma, dermatitis, rheumatoid arthritis, inflammatory bowel diseases and migraine are suggested to include a neurogenic component [3]. Many studies demonstrate that inflammatory peptides in a population of primary nociceptive neurons are critically important for induction and development of neurogenic inflammation. Experimentally, intradermal capsaicin (CAP) injection induces neurogenic inflammation and is characterized by arteriolar vasodilation, plasma extravasation, and pain (hyperalgesia and/or allodynia) [4-8]. The underlying mechanisms are that CAP sensitizes nociceptors by activating transient receptor potential vanilloid-1 (TRPV_1_) receptors distributed in small diameter myelinated (Aδ) and unmyelinated (C) primary afferent nociceptive fibers, which leads to the release of inflammatory peptides from these sensitized afferent terminals.

It is generally accepted that antidromic activation of afferent nociceptors is the cause of inflammatory peptide release and that dorsal root reflexes (DRRs) play a critical role in this process. DRRs are triggered pathophysiologically by excessive primary afferent depolarization of the central terminals in the spinal dorsal horn [9-11], which results from the opening of Cl^- ^channels and efflux of Cl^- ^ions from the synaptic terminals of primary afferents when GABA_A _receptors are activated by GABA released from spinal GABAergic interneurons [11,12]. DRRs are triggered in the spinal dorsal horn by GABAergic interneuronal circuits and conducted antidromically toward the periphery along the primary afferent nociceptive fibers [9,11,13-16].

Intradermal injection of CAP to activate TRPV_1 _receptors in primary afferent nociceptors can trigger and enhance DRRs [17,18], which are accompanied by flare (vasodilation) and edema (increased paw volume) in the paw [17,19], suggesting that there is a close relationship between enhanced DRRs and neurogenic inflammation presumably elicited by neuropeptide release [20]. The primary afferent fibers critically involved in triggering DRRs are CAP-sensitive fibers [18,21]. Although antidromic activation of primary nociceptive afferent endings (effector function) is well established to be a mechanism of driving the mediator release leading to neurogenic inflammation [22-26], there is no direct evidence to demonstrate that the release of inflammatory substances from nociceptive terminals is due to the triggering of DRRs and how activation of TRPV_1 _receptors initiates neurogenic inflammation via triggering DRRs. We hypothesize that the release of inflammatory peptides in the periphery is driven by the generation of DRRs, which contributes to the spread of cutaneous inflammation and to the development of neurogenic inflammation that exacerbates pain perception. This process is initiated by activation of TRPV_1 _receptors after CAP injection. To test this hypothesis, we have examined the role of the inflammatory neuropeptides, calcitonin gene-related peptide (CGRP) and substance P (SP), in DRR-mediated neurogenic inflammation by using the rat model of neurogenic inflammation induced by intradermal injection of CAP. Pharmacological and surgical manipulations were used to evaluate the role of DRRs [17,19]. The degree of acute cutaneous inflammation that followed intradermal injection of CAP was assessed by measurements of local blood flow (vasodilation) and paw-thickness (edema) of the rat foot skin. Some preliminary data have been presented in abstract form [27].

## Results

### Effects of dorsal root reflex removal on capsaicin- and neuropeptide-evoked inflammation

Observations on vasodilation and edema evoked by CAP and neuropeptides were made in three groups of rats for each agent.

#### Intradermal CAP-evoked inflammation

**Figure 1:**
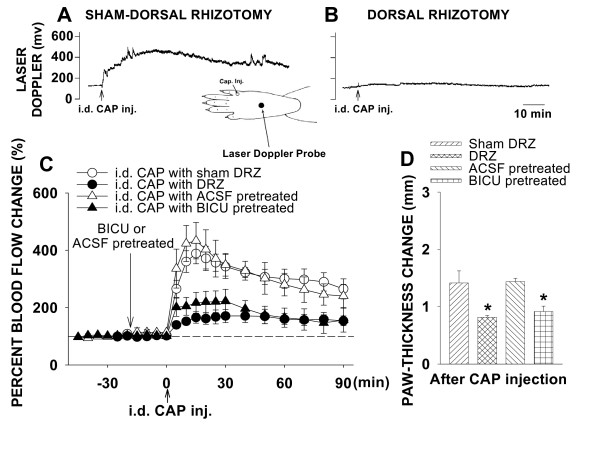
Changes in cutaneous blood flow and paw-thickness in the hindpaw of rats following ipsilateral intradermal (i.d.) injection of CAP in the hindpaw and the effects of dorsal rhizotomy (DRZ) and intrathecal bicucuclline (BICU). **A **and **B**: Samples of the laser Doppler flowmetry traces show changes in cutaneous blood flow in the rat hindpaw following CAP injection and the effects of dorsal rhizotomy. **C **and **D**: Mean results of blood flow and paw-thickness recordings summarizing the effects of DRZ and intrathecal BICU on the CAP-evoked inflammation. Blood flow pre-CAP injection was expressed as 100% (dashed line). Change in paw-thickness following CAP injection was presented as the difference score before and after CAP injection. Bicuculline (BICU) or ACSF was given intrathecally 20 min prior to CAP injection. Inset shows the sites where CAP was injected intradermally and blood flow was measured. *: P < 0.05, compared to the value in sham-dorsal rhizotomized or ACSP pretreated group.

In a group of rats (n = 7), the animals underwent sham surgery without sectioning the L_2_-S_1 _dorsal roots ipsilaterally. An elevated blood flow was seen at a site 15–20 mm away from the CAP injection spot (Fig. 1A) and reached its peak around 15 min after CAP injection (Fig. 1C). The peak increase and the value at 60 min after CAP injection were 388.7 ± 35.7% and 300.9 ± 33.3%, respectively (P = 0.0015 and P = 0.0023, compared with baseline level, one-way RM ANOVA; Fig. 1C). Change in paw-thickness on the side ipsilateral to CAP injection was presented as the difference score before and after CAP injection. In sham-operated group, the difference score of paw-thickness was 1.4 ± 0.2 (P = 0.003, compared with the group with intradermal vehicle injection, Dunnett's test; Fig. 1D). In the dorsal rhizotomized group of rats in which DRRs were removed surgically (n = 7), the enhanced blood flow induced by the same dose of CAP injected was much less than in rats with sham-dorsal rhizotomy (Fig. 1B). The blood flow increased slightly to 165.5 ± 19.9–171.1 ± 23.1% at 15–30 min after CAP injection and then recovered toward the baseline. Peak increase and the value at 60 min after CAP injection were 171.1 ± 23.1% and 159.9 ± 19.0%, respectively (P = 0.025 and P = 0.026, compared with baseline level, one-way RM ANOVA; Fig. 1C), which was much smaller than that in the sham-operated group (P = 0.0052 and P = 0.0063; Dunnett's test; Fig. 1B,C). The difference score of paw-thickness was significantly decreased to 0.82 ± 0.03 (P < 0.05, compared to the sham-operated group, Dunnett's test; Fig. 1D). Data from the group of rats in which DRRs were eliminated pharmacologically by pretreatment with bicuculline intrathecally (n = 7) show results similar to those of dorsal rhizotomized rats (Fig. 1C,D). Peak increase and the value at 60 min after CAP injection were 222.5 ± 41.6% and 164.9 ± 33.4%, respectively, which was much smaller than that in the intrathecal ACSF group (n = 6, P = 0.0082 and P = 0.032, Dunnett's test). Thus, the above data confirm that DRR removal led to an attenuation of the inflammatory reaction [17].

A control experiment has been done on the same model in our previous study by intradermal injection of vehicle (Tween 80 and saline), which did not produce obvious changes in blood flow and edema in the foot skin [17]. In addition, a previous study by our group showed that intradermal injection of CAP into the hindpaw did not significantly increase the blood flow level in the forepaw skin, suggesting that the local blood flow reaction is not the result of a change in systemic blood pressure [28].

#### Intra-arterial CGRP-evoked inflammation

**Figure 2:**
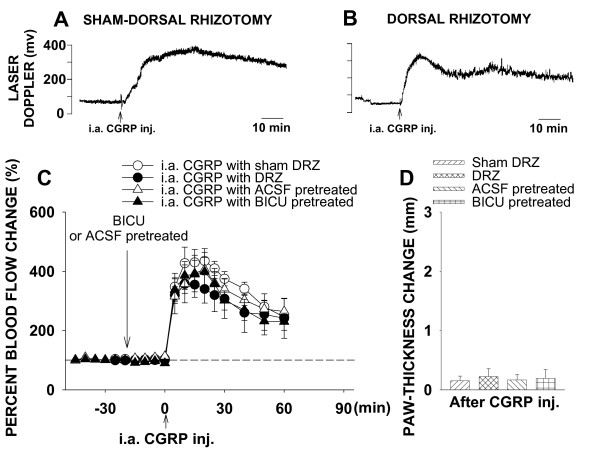
Changes in cutaneous blood flow and paw-thickness in the hindpaw of rats following ipsilateral intra-arterial (i.a.) injection of CGRP in the hindpaw and the effects of DRZ and intrathecal BICU. **A **and **B**: Samples of the laser Doppler flowmetry traces show changes in cutaneous blood flow of rat hindpaw following CGRP injection and the effects of DRZ. **C **and **D**: Mean results of blood flow and paw-thickness recordings summarizing the effects of DRZ and intrathecal BICU on the CGRP-evoked inflammation.

In a group of sham-dorsal rhizotomized rats (n = 6), local administration of CGRP by intra-arterial injection produced an increase in cutaneous blood flow in the hindpaw skin without significant change in the paw-thickness (Fig. 2). Blood flow level reached its peak at 20 min after CGRP application. The peak increase and the value of blood flow at 60 min after CGRP injection were 435.2 ± 41.4% and 245.8 ± 17.9%, respectively (P < 0.001 and P < 0.001, compared with baseline level, one-way RM ANOVA; Fig. 2C). However, removal of DRRs either surgically (dorsal rhizotomy, n = 7) or pharmacologically (intrathecal bicuculline, n = 7) produced no significant effects on the CGRP-evoked vasodilation and paw-thickness (Fig. 2B,C,D). In the dorsal rhizotomy group, P was 0.158 for the peak value when compared to sham group, and P was 0.181 for the value at 60 min after CAP injection when compared to sham group. In the intrathecal bicuculline group, P was 0.457 for the peak value when compared to intrathecal ACSF group (n = 6), and P was 0.825 for the value at 60 min after CAP injection when compared to intrathecal ACSF group. Difference score of paw-thickness in dorsal rhizotomized rats was 0.22 ± 0.13, P = 0.181, compared with sham-dorsal rhizotomized rats. Difference score of paw-thickness in the intrathecal bicuculline group was 0.19 ± 0.15, P = 0.198, compared with the intrathecal ACSF group.

#### Intra-arterial SP-evoked inflammation

**Figure 3:**
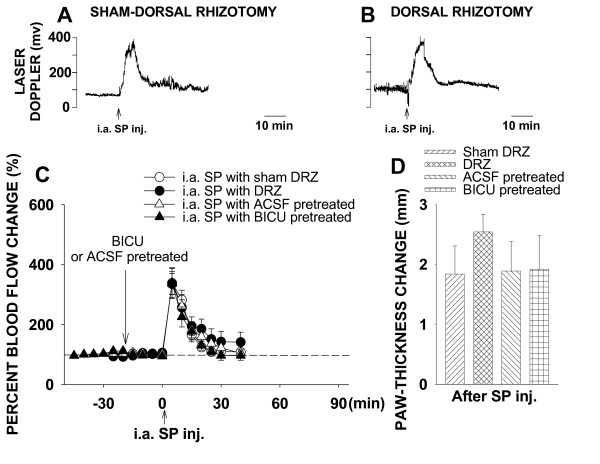
Changes in cutaneous blood flow and paw-thickness in the hindpaw of rats following ipsilateral i.a. injection of SP in the hindpaw and the effects of DRZ and intrathecal BICU. **A **and **B**: Samples of the laser Doppler flowmetry traces show changes in cutaneous blood flow of rat hindpaw following SP injection and the effects of DRZ. **C **and **D**: Mean results of blood flow and paw-thickness recordings summarizing the effects of DRZ and intrathecal BICU on the SP-evoked inflammation.

When dorsal roots were intact (sham-dorsal rhizotomy, n = 6), local administration of SP intra-arterially produced a short-lasting vasodilation and substantial edema (Fig. 3). Peak increase was 338.8 ± 38.8% at 5 min after SP application (P = 0.003, compared with the baseline value, one-way RM ANOVA). Consistent with the results of CGRP administration, neither surgical (dorsal rhizotomy, n = 6) or pharmacological (intrathecal bicuculline, n = 6) treatments affected significantly the SP-evoked inflammation (Fig. 3B, C, D). In the dorsal rhizotomy group, P was 0.954 for the peak value compared with the sham group. In the intrathecal bicuculline group, P was 0.879 for the peak value compared with the intrathecal ACSF group (n = 6). Difference score of paw-thickness in the dorsal rhizotomy group was 2.54 ± 0.29, P = 0.196, compared with the sham group. Difference score of paw-thickness in the intrathecal bicuculline group was 1.92 ± 0.56, P = 0.73, compared with the intrathecal ACSF group.

To exclude the possibility that the neuropeptide-evoked vasodilation was due to a systemic effect, change of blood flow in the forepaw was monitored simultaneously. The data show that local injection of these neuropepetides in the hindpaw did not produce significant change in blood flow in the forepaw (data not shown).

Thus, the differential effects of DRR removal on CAP- and neuropeptide-evoked inflammation indicate a close relationship between DRRs and the release of these neuropeptides.

### Effects of blockade of TRPV_1_, CGRP, neurokinin 1 or CGRP/neurokinin 1 receptors on the capsaicin-evoked inflammation

We further examined how the blockade of TRPV_1_, CGRP, neurokinin 1 (NK_1_), or CGRP/NK_1 _receptors in the periphery affected the CAP-evoked inflammation and what differences there were by using dose-response analyses of antagonistic effects.

#### Blockade of TRPV_1 _receptors by capsazepine

**Figure 4:**
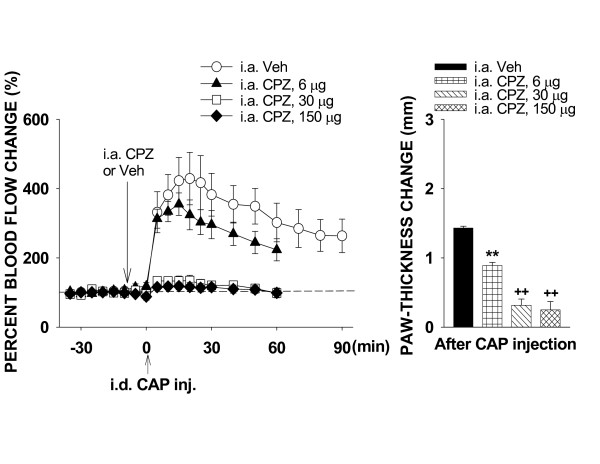
The effects of blockade of TRPV_1 _receptors on the CAP-evoked inflammation by pretreatment of the periphery with three different doses of capsazepine (CPZ). CPZ was given intra-arterially 10 min prior to CAP injection. **: P < 0.01, compared to the value in the group of i.a. injection of vehicle (Veh). **++**: P < 0.01, compared to the value with the lowest dose of the same drug.

After baseline measurements were taken, either capsazepine at one of three doses (6, 30 and 150 μg) or vehicle was given intra-arterially 10 min prior to CAP injection. The dose-response relationship (Fig. 4) shows that capsazepine produced a dose-dependent antagonism. A low dose (6 μg, n = 6) produced a slight reduction in the flare reaction (peak value was 355.0 ± 32.6%, P = 0.21, compared to the peak value, 429.0 ± 75.8%, in vehicle group, n = 6, Dunnett's test) and in the difference score of paw-thickness (0.89 ± 0.05, n = 6; P < 0.01, compared to the vehicle group, 1.4 ± 0.03, n = 6, Dunnett's test). When the periphery was pretreated with capsazepine at 30 or 150 μg, the inhibition of CAP-evoked inflammation reached a maximum (see Fig. 4). There was no statistical difference in the flare reaction or change in paw-thickness between groups receiving 30 μg (n = 7) or 150 μg (n = 7) of capsazepine. CAP-evoked flare was nearly completely abolished when the dose of capsazepine reached either 30 or 150 μg (Fig. 4). A comparison was further made between groups receiving 30 μg and 6 μg of capsazepine. In 30 μg group, the peak blood flow reaction and difference score of paw-thickness were 134.5 ± 15.4% and 0.36 ± 0.09, respectively, that were significantly lower than those in 6 μg group (P < 0.001 and P < 0.01).

#### Blockade of CGRP receptors by CGRP_8–37_

**Figure 5:**
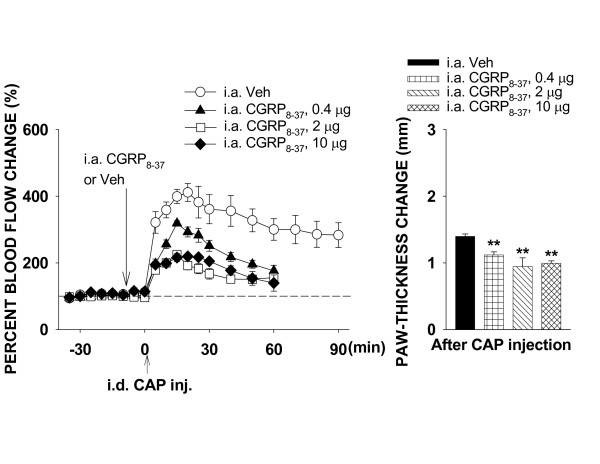
The effects of blockade of CGRP receptors on the CAP-evoked inflammation by pretreatment of the periphery with three different doses of CGRP_8–37_. CGRP_8–37 _was given intra-arterially 10 min prior to CAP injection. **: P < 0.01, compared to the value in the group of i.a. injection of vehicle (Veh).

The effect of blockade of CGRP receptors on the CAP-evoked inflammation was analyzed by pretreatment of the periphery with 3 doses of CGRP_8–37_. A dose-dependent inhibition of the CAP-evoked inflammation was seen with pretreatment with 0.4 (n = 6), 2 (n = 6) and 10 μg (n = 7) of CGRP_8–37_, respectively (Fig. 5). A slight decrease in flare reaction and paw-thickness change was induced by CAP injection when a low dose of CGRP_8–37 _(0.4 μg) was given (P < 0.01 and P < 0.01, compared to vehicle pretreatment group, n = 6, Dunnett's test). A further decrease in flare reaction and paw-thickness change was seen when the dose of CGRP_8–37 _reached either 2 or 10 μg. Unlike the blockade of TRPV_1 _receptors by capsazepine, blockade of CGRP receptors did not completely inhibit the flare and edema induced by CAP injection (Fig. 5). Since there was no statistical difference in the flare reaction and change in paw-thickness between groups receiving 2 μg or 10 μg of CGRP_8–37_, the inhibition of CAP-evoked inflammation by either 2 or 10 μg should presumably be maximal. A comparison was further made between groups receiving 2 μg and 0.4 μg of CGRP_8–37_. The peak blood flow reaction (225.1 ± 7.1%) in 2 μg group was significantly lower than that in the group receiving 0.4 μg of CGRP_8–37 _(P = 0.002). The difference score of paw-thickness in 2 μg group (0.95 ± 0.13) were slightly lower than that in the group receiving 0.4 μg of CGRP_8–37 _(1.12 ± 0.05), but the difference did not reach statistical significance (P = 0.246).

#### Blockade of NK_1 _receptors by spantide I

**Figure 6:**
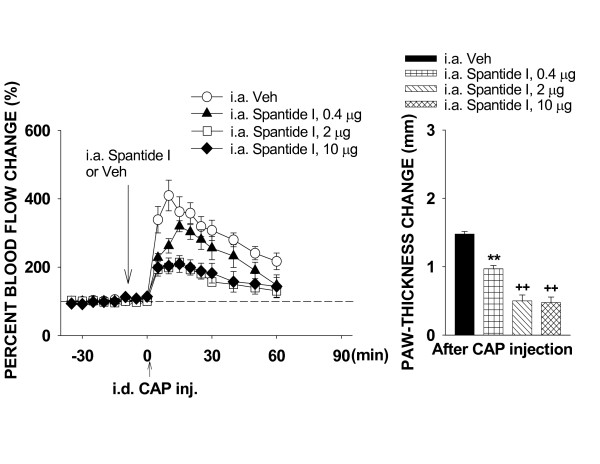
The effects of blockade of neurokinin 1 receptors on the CAP-evoked inflammation by pretreatment of the periphery with three different doses of spantide I. Spantide I was given intra-arterially 10 min prior to CAP injection. **: P < 0.01, compared to the value in the group of i.a. injection of vehicle (Veh). **++**: P < 0.01, compared to the value with the lowest dose of the same drug.

Similar to the results obtained from the experiments with CGRP_8–37_, a dose-dependent inhibition of CAP-evoked inflammation was seen with pretreatment with 0.4 (n = 6), 2 (n = 6) and 10 μg (n = 7) of spantide I, respectively (Fig. 6), but blockade of NK_1 _receptors did not completely inhibit the flare or edema induced by CAP injection. The inhibition of CAP-evoked inflammation by either 2 or 10 μg of spantide I should be maximal because there was no statistical difference in the flare reaction or change in paw-thickness between groups given 2 μg and 10 μg (Fig. 6). There was a significant difference both in peak reaction of blood flow and difference score of paw-thickness between groups receiving 2 μg and 0.4 μg of spantide I (P < 0.001 and P < 0.01). The blood flow reaction and difference score of paw-thickness were much lower in the group receiving 2 μg of spantide I.

**Figure 7:**
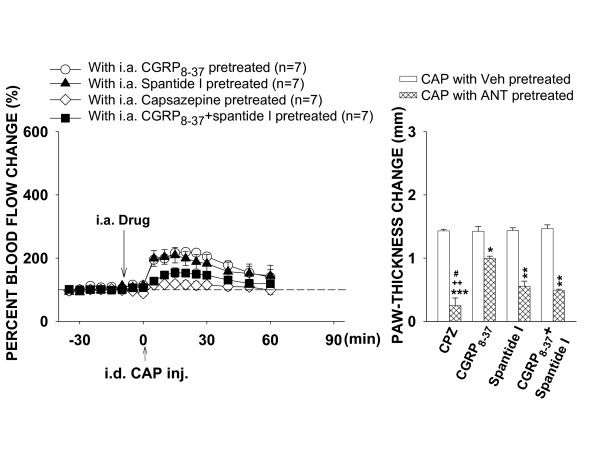
Summary of differential effects of blockade of peripheral TRPV_1_, CGRP, NK_1_, or CGRP/NK_1 _receptors on the CAP-evoked inflammation by intra-arterial injection of 150 μg capsazepine, 10 μg CGRP_8–37_, 10 μg spantide I, or co-administration of 10 μg CGRP_8–37 _and spantide I. *: P < 0.05, **: P < 0.01, ***: P < 0.001, compared to the value with vehicle pretreatment in the same antagonist (ANT) group. **++**: P < 0.01, compared to the value with CGRP_8–37 _pretreatment. **#**: P < 0.05, compared to the value with spantide I pretreatment.

Fig. 7 summarizes differential effects of blockade of TRPV_1_, CGRP, NK_1 _or CGRP/NK_1 _receptors on the CAP-evoked inflammation. The dose-response analyses of antagonism by the three antagonists have revealed that 150 μg of capsazepine, 10 μg of CGRP_8–37 _or spantide I produced a maximal inhibition of the CAP-evoked inflammation. Comparison of their inhibitory effects by these doses shows that vasodilation following CAP injection could be reduced by blockade of CGRP or NK_1 _receptors. The CAP-induced edema was also reduced by either blockade of CGRP or NK_1 _receptors, but the NK_1 _antagonist (spantide I) produced a much stronger inhibition of edema. However, when both CGRP and NK_1 _receptors were blocked by co-administration of 10 μg of CGRP_8–37 _and spantide I, inhibition of the CAP-evoked vasodilation became stronger. The peak value (154.1 ± 15.1%) was significantly lower than the peak value in the CGRP_8–37 _group (P = 0.035, Dunnett's test). Inhibition of the CAP-evoked edema by co-administration of CGRP_8–37 _and spantide I was slightly stronger compared to the group with spantide I pretreatment, but did not reach statistical significance. Finally, blockade of TRPV_1 _receptors abolished nearly completely the CAP-induced vasodilation and edema. The peak value of vasodilation in the capsazepine pretreated group was 118.3 ± 10.2%, which was statistically significant lower than that in CGRP_8–37 _pretreated (P = 0.001, Dunnett's test) and in spantide I pretreated (P = 0.005, Dunnett's test) groups, respectively, but not statistically significant lower than that in CGRP_8–37_+spantide I pretreated group (P = 0.073, Dunnett's test). Difference score of paw-thickness in the capsazepine pre-treated group was 0.25 ± 0.12, which was statistically significant smaller than that in CGRP_8–37 _pretreated (P < 0.01, Dunnett's test) and in spantide I pretreated (P < 0.05, Dunnett's test) groups, respectively, but not statistically significantly smaller than that in CGRP_8–37_+spantide I pretreated group (P = 0.164, Dunnett's test).

## Discussion

Previous studies by our and other groups on an acute experimental model of neurogenic inflammation evoked by intradermal injection of CAP have physiologically and pharmacologically demonstrated that cutaneous inflammatory reactions characterized by local vasodilation (flare) and edema (increased paw-thickness) are predominantly mediated by triggering DRRs [17,19,20]. DRR activity has been recorded electrophysiologically from the central end of individual Aδ- and C-primary afferents and shown to be enhanced after CAP injection [18,21,29]. In the present study, we have further extended our ongoing project in the following respects. **1) **New evidence has been provided to confirm the view that DRRs are triggered and then enhanced by activation of TRPV_1 _receptors to evoke neurogenic inflammation by driving the release of neuropeptides (CGRP and/or SP). **2) **pharmacological studies using dose-response analyses of antagonism of TRPV_1 _and neuropeptide receptors reveal that the released CGRP and SP participate critically in the neurogenic inflammation; **3) **activation of TRPV_1 _receptors in primary afferent nociceptors following CAP injection initiates this process, including triggering of DRRs.

Many primary nociceptive afferent neurons and their axons (Aδ- and C-fibers) are peptidergic with the capacity to release inflammatory peptides [30-35]. CGRP and SP are major inflammatory mediators that contribute a neurogenic component to inflammation [36,37]. When released from primary afferent neurons, CGRP and SP produce neurogenic inflammation by interacting with endothelial cells, mast cells, immune cells and arterioles. For instance, CGRP is potent vasodilator that produces a strong and long-lasting vasodilation [38], and SP results preferentially in stronger plasma extravasation [39].

A critical concern addressed in the present study is the mechanism by which inflammatory mediators are released in the periphery to induce neurogenic inflammation. It has been suggested that intradermal injection of CAP results in a local vasodilation, increased plasma extravasation, and hyperalgesia through release of neuropeptides from peripheral primary afferent terminals [11,40-42]. These afferent fibers can be sensitized by CAP due to activation of TRPV_1 _receptors, a key nociceptive molecule expressed in these fibers, to contribute to nociceptive transmission and neurogenic inflammation [5,6,43-45]. Thus, CAP plays not only a sensory role by activating nociceptors, but it also has an efferent function by initiating neurogenic inflammation. The latter results from CAP-induced Ca^2+ ^influx into nerve terminals through TRPV_1 _receptors and voltage-dependent Ca^2+ ^channels, causing the exocytosis of inflammatory mediators [46-49] and their release into the periphery to produce sensitization of primary afferent nociceptors and neurogenic inflammation [50-53]. The above process can be modulated by antidromic activation of afferent fibers, which would drive and trigger the release of inflammatory mediators that initiates neurogenic inflammation, because experimentally antidromic activation of the cut dorsal roots can evoke obvious vasodilation and plasma extravasation when the electrical stimulus strength is strong enough to active C-fibers [54-56]. In the present study, experiments were designed to determine whether there was a release of CGRP and SP from sensory afferent terminals (nociceptors) and whether this release was antidromically driven by DRRs in the CAP-evoked neurogenic inflammation. We proposed that removal of DRRs would interrupt this pathway to alleviate the neurogenic inflammation induced by CAP injection. The data have shown that local vasodilation and increased paw-thickness evoked by CAP injection were greatly reduced after dorsal rhizotomy or intrathecal bicuculline administration that removed DRRs. In contrast, inflammatory reactions evoked by direct application of CGRP or SP in the periphery that would mimic the DRR-mediated inflammation induced by CAP injection were unchanged under the same conditions when DRRs were removed. Thus, there should be a close relationship between DRRs and the release of these neuropeptides based on the observations of differential effects of DRR removal on CAP- and neuropeptide-evoked inflammation, which suggests that the release of CGRP and/or SP is driven by DRRs to participate critically in the CAP-evoked inflammation. In this process, activation of TRPV_1 _receptors appears to be an initial step. Therefore, we wanted to analyze further how neurogenic inflammation was initiated and developed via DRRs by differentiating the roles of TRPV_1_, CGRP and NK_1 _receptors.

Dose-response analysis of the antagonistic effect of the TRPV_1 _receptor antagonist, capzasepine, on the CAP-evoked inflammation indicates that vasodilation and edema evoked by CAP injection are inhibited in a dose-dependent manner by capsazepine pretreatment. When the dose of capsazepine was in the range of 30–150 μg, the inhibition seemed to reach a maximum. This result is consistent with studies on other pain models that a blockade of TRPV_1 _receptors by similar doses of capsazepine antagonized selectively the CAP-evoked hyperalgesia and alleviated other inflammogen-evoked pain behaviors in a dose-dependent manner [57-59]. Importantly, CAP-evoked inflammation was nearly completed blocked with these doses. This suggests that neurogenic inflammation after CAP injection is initiated by activation of TRPV_1 _receptors that in turn trigger and then enhance DRRs, which release inflammatory neuropeptides.

Since the mechanism underlying neurogenic inflammation evoked by CAP injection and driven by DRRs seems to be the result of CGRP and/or SP release, we assumed that a blockade of either CGRP or NK_1 _receptors in the periphery should alleviate the inflammation. The analysis of antagonistic effects of blockade of CGRP or NK_1 _receptors by examining the dose-response relationships when CGRP_8–37 _or spantide I was given as a pretreatment shows that each antagonist when given individually reduced the CAP-evoked inflammation in a dose-dependent manner, but the inflammation was not completely abolished when the effect of each antagonist was maximal. Thus, each neuropeptide released contributes partially to neurogenic inflammation initiated by CAP injection via activation of TRPV_1 _receptors. A further analysis of blockade of both CGRP and NK_1 _receptors revealed that the CAP-evoked inflammation (prominently vasodilation) was more effectively alleviated by co-administration of CGRP_8–37 _and spantide I compared to the effect of a single antagonist. This suggests that CGRP and SP are two major inflammatory mediators in the neurogenic inflammation initiated by activation of TRPV_1 _receptors and driven by triggering of DRRs.

In summary, the present results update the role of DRRs in neurogenic inflammation by providing new evidence to suggest that the release of CGRP and SP in the periphery is driven by the generation of DRRs, which participate critically in neurogenic inflammation with that pain perception is exacerbated. Further, this process is initiated by activation of TRPV_1 _receptors after CAP injection.

## Methods

### Experimental animals

Male Sprague-Dawley rats weighing 250–350 g were used in this study. The animals were housed in groups of two to three, with food and water available *ad libitum*, and were allowed to acclimate under a light/dark cycle for approximately 1 wk prior to experiments. The experiments were carried out in accordance with the National Institutes of Health Guide for the Care and Use of Laboratory Animals and with the approval of the Institutional Animal Care and Use Committee of the University of Texas Medical Branch. All efforts were made to minimize the number of animals used and their suffering.

Rats were initially anesthetized with sodium pentobarbital (i.p. 50 mg kg^-1^) to perform surgery. Anesthesia was then maintained throughout the experiment by continuous intravenous infusion of a saline solution containing sodium pentobarbital. The infusion rate was adjusted (5–8 mg kg^-1 ^h^-1^) depending upon the depth of anesthesia. The depth of anesthesia was judged as being sufficiently deep when withdrawal responses to noxious limb stimulation and/or the eye-blink reflex to air-puffs were absent. Once anesthetic level was adequately established, the animals were paralyzed with pancuronium (0.3–0.4 mg h^-1^, i.v.). The rats were then ventilated artificially, and end-tidal CO_2 _was physiologically kept between 3.5 and 4.5% by adjusting the respiratory parameters. The adequacy of the depth of anesthesia during an experiment was evaluated by the examination of the pupillary reflexes and assessing the stability of the expired CO_2_. Cutaneous blood flow and paw-thickness were measured on anesthetized and paralyzed rats because a series of previous studies on blood flow measurements by our group have been conducted under the same conditions [17,28,60,61]. Rectal temperature was monitored using a rectal probe and maintained at 37°C by a servo-controlled heating blanket.

### Induction of acute cutaneous inflammation

An acute cutaneous inflammation model was induced by intradermal injection of CAP (from Fluka, prepared in a solution of 7% Tween 80 and 93% saline at a concentration of 1%) as previously described [17,28,61]. CAP was injected intradermally into the plantar surface of the foot in a volume of 15 μl. Control experiments were done by vehicle injections using Tween 80 and saline at the same volume as the CAP solution [17].

### Measurements of cutaneous vasodilation

Blood flow was detected as blood cell flux by a laser Doppler flowmeter (Moor Instruments, UK). The output showing blood flow level was then recorded by a computer data acquisition system (CED 1401 plus, with Spike-2 software) in millivolt units (see panels **A **&**B **in Figs. 1, 2 and 3) and also in [17,19,28,60,61]. To measure the cutaneous blood flow level and the local vasodilation (flare) that followed intradermal injection of CAP into the skin of the foot, the probe from the laser Doppler flow meter was attached to the plantar skin surface of the foot with adhesive tape. The flowmeter we used has been reported to produce a laser beam that penetrates to a depth of 500–700 μm below the surface where the probe is placed [62], which assured that the laser Doppler flow probe picked up the blood flow signal mainly from the microvasculature in the dermis.

The flare reaction after CAP injection could be detected at distances up to 30 mm away from the CAP injection spot. A number of studies by our group [17,19,28,60,61] have consistently indicated that a large blood flow reaction seen at a distance of 15–20 mm away from the site where CAP was injected is mainly mediated by DRRs. In this study, therefore, we only measured the blood flow changes in the foot skin at a distance of 15–20 mm away from the CAP injection spot (see inset in Fig. 1).

### Paw-thickness measurements

The degree of cutaneous inflammation due to CAP injection was also assessed by paw-thickness measurements to reflect edema due to plasma extravasation. This was done with a digital caliper placed near the site where the laser Doppler probe was placed. Care was taken to assure that the caliper was placed at the same site on the paw for each measurement. Each measurement was the mean value calculated from 3 trials [17].

### Surgical and pharmacological elimination of DRRs

To evaluate the involvement of DRRs in driving the release of CGRP and/or SP to contribute to neurogenic inflammation, inflammation was evoked under conditions that DRRs were eliminated surgically or pharmacologically.

#### Dorsal rhizotomy

This was done to eliminate DRRs surgically. Laminectomy was performed to expose the dorsal roots of segments L_3_-S_1 _bilaterally. The exposed cord and roots were protected from drying and cooling by formation of warmed oil pool between skin flaps. The dorsal roots that needed to be sectioned were gently dissected, and a small piece of cotton containing 2% lidocaine was applied to them at the site where the roots were to be cut to minimize injury discharges.

#### Intrathecal administration of bicuculline

This was done to eliminate DRRs pharmacologically [12,17,63,64]. The suboccipital region was exposed by a midline incision; the dura over the cisterna magna was opened with a small vertical incision, and a catheter (32G, from Micor, Allison Park) was advanced through a guide cannula to the spinal subarachnoid space at the T_12_-L_1 _vertebral level for intrathecal administration. Five μg of bicuculline (a GABA_A _receptor antagonist from Sigma-Aldrich) dissolved in artificial cerebrospinal fluid (ACSF) in a volume of 15 μl, was injected intrathecally. A previous study has demonstrated that 5 μg of bicuculline administered intrathecally can effectively block DRRs and CAP-evoked inflammation [17,60].

### Peripheral administration of agonists and antagonists of inflammatory peptide receptors and antagonist of TRPV_1 _receptors

Close-by intra-arterial injections were used to deliver drugs to the periphery [28,29,45,61]. To do this, one branch of the femoral artery on the side of blood flow measurement was carefully isolated from connective tissue and ligated proximally. The artery was then cannulated distally by a small sized polyethylene tube that was connected with a Hamilton syringe. Drugs were given intra-arterially in a volume of 10 μl.

#### Experimental protocol

1. To determine whether the release of CGRP or SP from sensory afferent terminals (nociceptors) was driven by DRRs and the role in the CAP-evoked neurogenic inflammation, the spread of flare and edema in the plantar skin of the foot on the side ipsilateral to local injection of CAP (1%, 15 μl), CGRP (from Tocris, 1.0 μg) or SP (from Tocris, 0.1 μg) were measured. CAP was injected intradermally, and CGRP or SP was injected intra-arterially. Solutions of CGRP and SP were made with saline (pH corrected to 7.2–7.4). After local injection of CAP, CGRP or SP, changes in blood flow and paw thickness were recorded and monitored for 1–1.5 hr, and the effects were compared to the effects of the same agents evoked under the conditions when DRRs were removed surgically (dorsal rhizotomy) or pharmacologically (intrathecal administration of bicuculline at a dose of 5 μg). Dorsal rhizotomy (L_2_-S_1_) was performed on the side ipsilateral to the injection on the day when the experiment was conducted.

2. The effects of blockade of TRPV_1_, CGRP, NK_1_, or both CGRP and NK_1 _receptors on the CAP-evoked neurogenic inflammation were analyzed pharmacologically. After control values of blood flow and paw thickness were recorded, three doses of each antagonist (capsazepine, CGRP_8–37_, or spantide I) were given intra-arterially in different groups of animals 10 min prior to CAP injection. These included the TRPV_1 _receptor antagonist, capzazepine (from Tocris) at doses of 6, 30 and 150 μg [57-59]; the CGRP receptor antagonist, CGRP_8–37 _(from Tocris), at doses of 0.4, 2.0 and 10.0 μg [65,66] and the NK_1 _receptor antagonist, spantide I (from Tocris) at doses of 0.4, 2.0 and 10.0 μg [67,68]. Capsazepine was dissolved in vehicle made from 10% DMSO and 90% saline. CGRP_8–37 _and spantide I were dissolved in saline. The changes both in blood flow and paw thickness were monitored for 1–1.5 h following CAP injection. The inhibition of the CAP-evoked inflammation induced by the highest dose of each antagonist or co-administration of CGRP and NK_1 _receptor antagonists were compared among groups of capsazepine, CGRP_8–37_, spantide I and CGRP_8–37_/spantide I pretreated animals. In separate groups, vehicle used for making the solution of each antagonist was injected prior to CAP injection for control purposes.

### Data analysis

All data are expressed as mean ± S.E. Baseline blood flow level (pre-CAP) was expressed as 100% and percentage changes after CAP injection were compared for groups of animals that received different treatments. A change in paw-thickness following CAP injection is presented as the difference score before and after CAP injection and compared for the groups of animals that received different treatments. Statistical differences between groups were determined by one-way ANOVA followed by the Dunnett's analysis. Data obtained before and different time points after CAP injection were compared using one-way repeated measures ANOVA followed by Student t-tests. *P *< 0.05 was considered statistically significant.

## Abbreviations

ACSF, artificial cerebrospinal fluid; ANOVA, analysis of variance; BICU, bicuculline; CGRP, calcitonin gene-related peptide; CAP, capsaicin; CPZ, capsazepine; DMSO, dimethyl sulfoxide; DRRs, dorsal root reflexes; DRZ, dorsal rhizotomy; NK_1_, neurokinin 1; SP, substance P; TRPV_1_, transient receptor potential vanilloid-1.

## Competing interests

The author(s) delare that they have no competing interests.

## Authors' contributions

QL conceived and designed the experiments, and prepared the manuscript. DL, XX, XZ and QL performed experiments. LF participated in finalizing manuscript. All authors read and approved the final manuscript.
